# Individual Cases

**Published:** 1918-01

**Authors:** 


### *Individual Cases.*

Espitalier and Vitoux report through P. Bazy (*Bulletins et Mémoires*, March 6, 1917, p. 540) a case in which late tetanus developed in a patient whose wound, received 4 months previously, had healed. The tetanus followed in 6 days after a reopening of the wound for the removal of a piece of shell. 10 cc. of serum were injected. Death occurred 2 days later, although the temperature was never higher than 37.6° C. The patient had probably had a preventive injection at the time of wounding. The authors recommend a preventive injection before all operations upon men who have previously been wounded in war.

Phelip and Policard report through Broca (*Bulletins et Mémoires*, May 11, 1915, p. 1007) a case in which neuritis in the right arm seems to have prepared the way for an attack of tetanus announced by local spasms. The tetanus, in spite of 2 preventive injections within 24 hours of a slight wound in the palm of the right hand, developed 17 days after the wound. The case was treated also curatively by injections of 20 cc. intrathecally and 10 cc. subcutaneously. Recovery resulted within 3 weeks.

Mouat (in the *Lancet*, Jan. 22, 1916) reports a case in which tetanus appeared in the form of local twitchings which were first ascribed to ascending neuritis. During 23 days before an amputation was thought necessary, the patient had complained of painful spasms in the injured limb. These had been attributed also to neuritis. Two days after the operation the twitchings were renewed and on the 8th day the flaps of the stump were opened up. At the first operation it had been noted that the nerves were swollen, and that they stood out in a striking manner on the face of the stump. They had been pulled down and cut off. On the 11th day the patient developed trismus. 3,000 U. S. A. units were administered intrathecally and 1,500 were also injected in or about the upper cords of the brachial plexus on the left side. The symptoms grew worse. A few days later a second intrathecal injection of 6,000 units seemed to improve the case, which was on the road to complete recovery. Death, however, occurred, apparently as the result of a subdiaphragmatic abscess. M. calls especial attention to the nature of the onset in muscular spasms, and the apparent effectiveness of the doubled dose of antitoxin administered intrathecally.

Penhallow (*Lancet*, Feb. 26, 1916) reports a case in which tetanus developed in wounds that had healed, 2 months after the injury, apparently as the result of an attempt under ether to overcome contracture of the forearm and to correct the position of the leg. In all 6,000 units of serum were administered, and the patient recovered. The serum seemed to be very effective, although it was administered subcutaneously and intravenously in comparatively small doses of 1,500 units.

Mullaly (*Lancet*, April 22, 1916) reports a case in which twitchings similar to those described by Mouat followed amputation for serious wounds of the leg, complicated with gaseous gangrene. M. attributed the twitchings to ascending neuritis, and attempted to improve the condition by cutting away as much of the swollen nerves as he could reach. The patient died without any general symptoms of tetanus.

Foster (*Brit. Med. Jour.*, Feb. 10, 1917, p. 189) reports 3 cases of late tetanus that developed in wounds not obviously septic. The tetanus had lain dormant for a considerable period (146, 106, and 86 days). In 2 cases a fall seems to have brought on the tetanus symptoms; in the third case there was no apparent cause for the onset. All three cases concerned fractures that had been healed. F. notes that in all the period of immunity conferred by preventive injections had passed. He concludes that repeated prophylactic injections should be given in all cases of gunshot wounds whatever the signs of infection.

Westwater (*Brit. Med. Jour.*, March 24, 1917, p. 395) reports a case of recurrent tetanus after a first attack of tetanus of unusual severity, which apparently had been overcome by an intrathecal administration of 27,000 units. During the period of recovery from the first attack, preventive doses of 500 units were given twice in 40 days. At the end of this time a second severe attack of tetanus developed, and the patient died in spite of 21,000 units administered intrathecally.

